# CAR T Cell Therapy in Lung Cancer: Overcoming Tumor Microenvironment-Mediated Barriers

**DOI:** 10.7150/ijbs.131261

**Published:** 2026-07-22

**Authors:** Lu Liu, Dandan Liang, Cui Wang, Jian Li, Jie Tang, Weimin Li, Xijie Yu

**Affiliations:** 1 Laboratory of Endocrinology and Metabolism, Department of Endocrinology and Metabolism, West China Hospital, Sichuan University, Chengdu, China.; 2 Institute of Respiratory Health, Frontiers Science Center for Disease-related Molecular Network, West China Hospital, Sichuan University, Chengdu, China.; 3 State Key Laboratory of Respiratory Health and Multimorbidity, West China Hospital, Chengdu, China.; 4 Department of Endocrinology and Metabolism, Shandong Second Provincial General Hospital, No. 4, Duanxing West Road, Jinan, Shandong Province, China.

**Keywords:** lung cancer, CAR T cell therapy, tumor microenvironment, immunotherapy, solid tumors

## Abstract

Chimeric antigen receptor (CAR) T cell therapy has achieved remarkable clinical success in hematological malignancies. However, its efficacy in solid tumors such as lung cancer remains constrained by the immunosuppressive tumor microenvironment (TME). Aberrant vascular architecture and dense stroma constitute major physical barriers that hinder CAR T cell infiltration. Additionally, an immunosuppressive cellular network, dominated by myeloid-derived suppressor cells and tumor-associated macrophages, further restricts CAR T cell expansion and function. Moreover, immune checkpoint signaling, inhibitory cytokines, dysregulated chemokine gradients, and metabolic reprogramming under hypoxia collectively create a hostile biochemical and metabolic milieu that drives CAR T cell dysfunction and exhaustion. This review systematically outlines these multifactorial barriers within the lung cancer TME and discusses emerging strategies, including combinatorial approaches, engineered CAR T designs, and microenvironment-modulating platforms, that aim to improve the therapeutic efficacy of CAR T cell therapy in lung cancer.

## 1. Introduction

Lung cancer remains one of the leading causes of cancer mortality worldwide. In 2022, it accounted for approximately 2.48 million new cases and 1.82 million deaths globally 1. Targeted therapies and immune checkpoint blockade have reshaped the treatment landscape, and programmed cell death protein 1 (PD-1) or programmed death-ligand 1 (PD-L1) inhibitors are now incorporated into first-line regimens for advanced non-small cell lung cancer (NSCLC). However, durable benefit remains limited to a subset of patients. In extensive-stage small cell lung cancer (SCLC), etoposide-platinum chemotherapy yields a median overall survival of about 10 months, and the addition of atezolizumab extends survival to 12.3 months 2. Against this backdrop, chimeric antigen receptor (CAR) T cell therapy, which involves engineering autologous T cells to recognize specific tumor antigens, has achieved transformative success in hematological malignancies. To date, the U.S. Food and Drug Administration (FDA) has approved multiple CAR T cell products, which in relapsed or refractory blood cancers can induce overall response rates typically in the range of 70-90%, with a subset of patients achieving remissions lasting for years 3,4. This success underscores the transformative potential of cellular immunotherapy.

However, in solid tumors, including lung cancer, the clinical activity of CAR T cell therapy remains markedly lower than that achieved in hematologic malignancies 5-8. This efficacy gap indicates that the limitations of CAR T therapy in solid tumors extend beyond antigen recognition itself. In lung cancer, multiple barriers coexist and reinforce one another, including heterogeneous and dynamic target antigen expression, a narrow therapeutic window caused by on-target/off-tumor toxicity, insufficient tumor homing and infiltration, limited *in vivo* expansion and persistence, and a highly immunosuppressive tumor microenvironment (TME). Thus, target expression does not directly translate into durable efficacy. Even when CAR T cells can recognize tumor cells, their ability to enter tumor tissue, maintain effector function, and resist local suppressive signals remains decisive for therapeutic outcome.

The solid tumor TME is a complex and dynamic ecosystem composed of malignant cells, immune cells, stromal cells, vascular cells, extracellular matrix (ECM), soluble mediators, and metabolic signals. Aberrant vascular architecture and dense fibrotic ECM form prominent physical barriers that impede immune cell trafficking and restrict CAR T cell entry into the tumor parenchyma. Meanwhile, the TME also establishes a suppressive biochemical and metabolic environment, including hypoxia, extracellular acidification, nutrient competition, and increased reactive oxygen species. Immunosuppressive mediators such as TGF-β, PGE2, adenosine, and IL-10 are also frequently elevated, accompanied by the accumulation of regulatory T cells, myeloid-derived suppressor cells, and tumor-associated macrophages 9,10.

These barriers do not act independently, but converge to impair CAR T cell activation, metabolic fitness, and persistence *in vivo*. Under persistent antigen stimulation and stress derived from the TME, CAR T cells may gradually acquire an exhausted phenotype, characterized by reduced proliferation and cytotoxicity, decreased cytokine production, and sustained expression of inhibitory receptors such as PD-1, LAG-3, TIM-3, and TIGIT 11. This pressure is particularly relevant in lung cancer, where extensive stromal remodeling, hypoxia, immune exclusion, and treatment-induced changes in the microenvironment can simultaneously limit CAR T cell access and suppress effector function 9,10. Thus, lung cancer represents a solid tumor context in which antigenic, structural, cellular, and metabolic barriers are highly intertwined.

Before discussing these TME-mediated barriers in detail, this review first outlines the current landscape of CAR T cell therapy in lung cancer, including target selection, early clinical evidence, antigen heterogeneity, safety constraints, emerging CAR T design strategies, and key translational challenges. We then examine the physical, cellular, biochemical, and metabolic barriers imposed by the lung cancer TME and summarize corresponding combination strategies, engineered CAR T designs, and approaches that modulate the microenvironment.

## 2. Current landscape of CAR T cell therapy in lung cancer

CAR T cell therapy in lung cancer remains at an early stage of clinical translation. Although preclinical pipelines continue to expand and the number of clinical trials is increasing, current studies are still largely small scale, early phase, and exploratory. Ongoing research covers both NSCLC and SCLC and targets a range of tumor-associated antigens (TAAs) (Table 1). However, the growing number of candidate targets has not yet translated into durable and reproducible clinical benefit. This gap suggests that the translational challenges of lung cancer CAR T therapy cannot be explained by insufficient antigen recognition alone, but should be understood across multiple dimensions, including target stability, therapeutic window, adaptation to the TME, and product persistence.

### 2.1 Target selection, therapeutic window, and antigen escape

One of the central challenges in lung cancer CAR T development is the selection of target antigens with sufficient tumor selectivity and relatively stable expression. Many candidate antigens are expressed in lung cancer, but they can also be detected at varying levels in normal tissues or inflammatory settings, thereby narrowing the therapeutic window and increasing the risk of on-target/off-tumor toxicity. Therefore, target selection should not be based solely on tumor expression frequency. It should also consider normal tissue distribution, differences in antigen density, expression stability under therapeutic pressure, and compatibility with CAR designs that incorporate safety control.

PD-L1 is a representative example of a target with a narrow therapeutic window. It functions as an immunosuppressive checkpoint ligand in the lung cancer TME, but it has also been explored as a potential CAR T target or engineering module 45. However, PD-L1 is not specific to tumors, and its expression in normal tissues or inflammation-associated cells may increase the risk of on-target/off-tumor toxicity. Indeed, a phase I clinical trial evaluating CAR T cells targeting PD-L1 in lung cancer (NCT03330834) was terminated early because of serious adverse events. These observations suggest that PD-L1-directed CAR T cells require additional safety designs, such as affinity tuning, logic gating, switchable control, or local delivery.

Beyond the therapeutic window, antigen heterogeneity and antigen escape are major limitations of CAR designs with fixed single-antigen specificity. Lung cancer exhibits marked spatial and temporal heterogeneity, reflected by regional differences in antigen expression and dynamic changes in target abundance under therapeutic pressure. CAR T cells targeting a single antigen may preferentially eliminate tumor subclones with high antigen expression, whereas antigen-low or antigen-negative populations may gain a selective advantage and drive relapse. In SCLC, transcriptional programs defined by ASCL1, NEUROD1, POU2F3, and inflammatory features may further influence immune sensitivity and target antigen expression, thereby reducing the stability of fixed single-target designs 46,47.

Accordingly, expanding antigen coverage while improving safety control has become an important direction in lung cancer CAR T design. Bispecific CARs, tandem CARs, and dual CARs mainly reduce the risk of escape from a single antigen by broadening antigen recognition. Logic-gated CARs and affinity tuning place greater emphasis on tumor selectivity, aiming to reduce injury to normal tissues with low target antigen expression. Switchable systems and adapter-dependent or modular CAR platforms further separate antigen recognition from CAR signaling activation, allowing CAR T activity to be titrated, paused, or redirected by adjusting adapter selection, dose, administration schedule, or withdrawal 37,48-53. Representative strategies include dual targeting of MUC1 and PSCA in NSCLC models, dabrafenib-mediated upregulation of MUC1 to enhance targetability, and the ongoing EGFR/B7-H3 bispecific CAR T trial (NCT05341492) 20,23,54. However, design with multiple targets is not simply a matter of adding more targets; it may also increase structural complexity, nonspecific activation, and safety risks. Therefore, heterogeneity-tolerant CAR T designs should be developed together with safety control mechanisms, rather than pursuing broader antigen coverage alone.

### 2.2 Clinical evidence and translation

Current clinical evidence for CAR T therapy in lung cancer remains preliminary. Studies differ substantially in target selection, CAR structure, conditioning regimen, delivery route, enrolled population, and clinical endpoints, making direct comparisons across studies difficult. Therefore, available clinical data mainly reflect the initial feasibility and safety boundaries of CAR T therapy in lung cancer, but are still insufficient to establish stable and durable efficacy. **Table 1** systematically summarizes representative targets, preclinical and clinical evidence, and key translational issues in lung cancer CAR T therapy, providing an overview from target discovery to clinical application.

From a translational perspective, the current gap is not a lack of candidate targets, but the failure to consistently convert these targets into durable therapeutic benefit. Most early studies have enrolled patients with advanced, heavily pretreated disease, who often have high tumor burden, rapid disease progression, and impaired T cell quality. These factors may compromise autologous CAR T manufacturing quality, *in vivo* expansion, and persistence 11. Meanwhile, spatial exclusion, immunosuppression, and metabolic stress within the lung cancer TME may further limit the ability of CAR T cells to maintain effector function after entering tumors. Therefore, the durability of efficacy, optimal patient selection, and best combination strategies for lung cancer CAR T therapy remain to be further defined.

Overall, translational optimization of lung cancer CAR T therapy should not be limited to “selecting better targets” or “building stronger CARs” as separate goals. Instead, target coverage, safety control, tumor accessibility, and functional adaptability of CAR T cells should be integrated into a unified design framework. The following sections further discuss the physical, cellular, biochemical, and metabolic barriers that affect CAR T cell trafficking, survival, and effector function in the lung cancer TME, and map corresponding engineering and combination strategies to these resistance mechanisms.

## 3. Physical barriers in the TME

**Figure 1:**
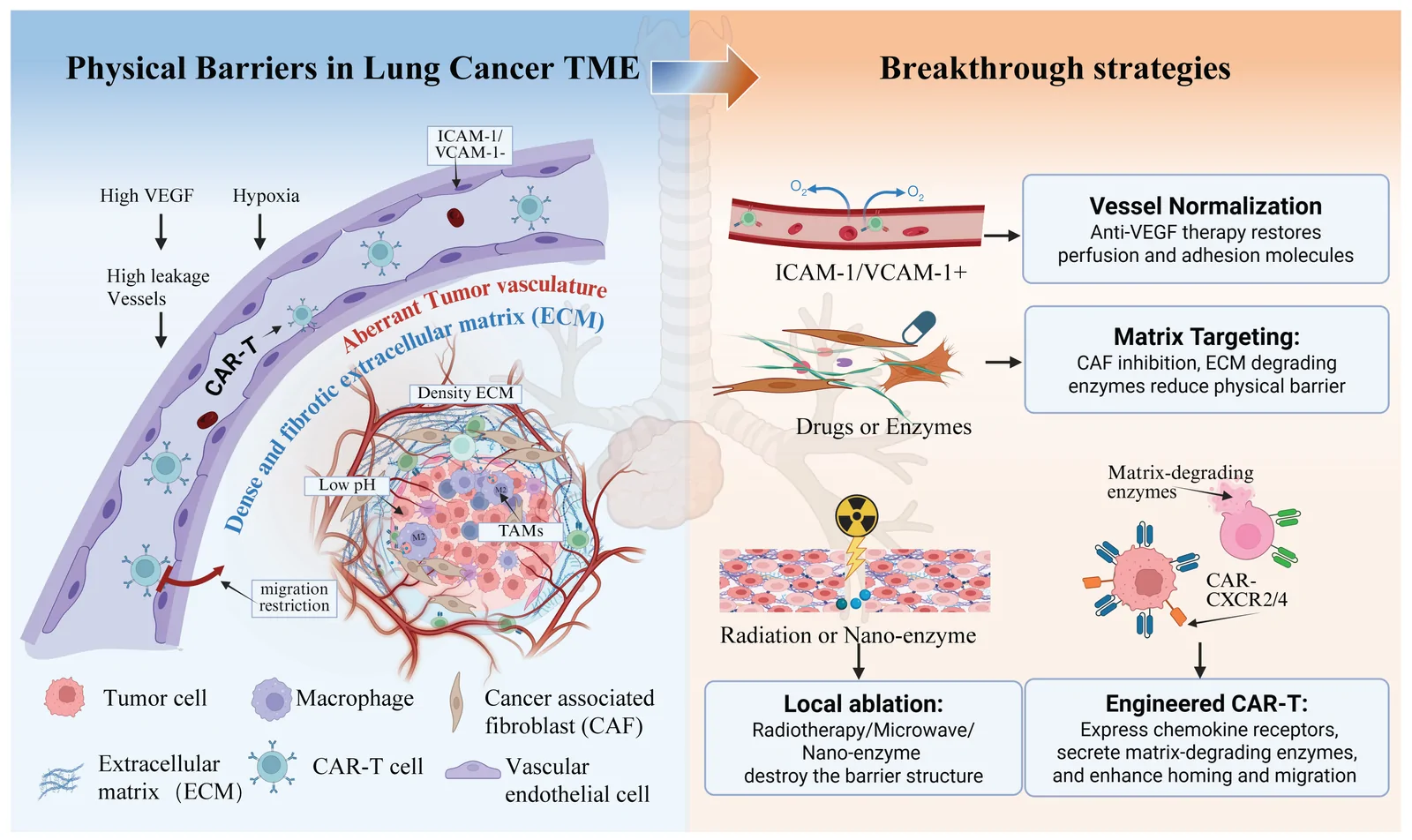
** Physical barriers and intervention strategies for CAR T cell access in lung cancer.** Schematic illustration of physical barriers in the lung cancer tumor microenvironment and breakthrough strategies to enhance CAR T cell access, highlighting aberrant tumor vasculature and dense, CAF-shaped extracellular matrix that restrict extravasation and interstitial migration, and corresponding interventions including vessel normalization, matrix targeting, local ablation, and engineered CAR T cell designs that improve homing and tissue penetration (Created with BioRender.com).

In solid tumors, physical barriers represent an early and often decisive constraint on CAR T cell efficacy. Following systemic infusion, CAR T cells must first traverse abnormal tumor vasculature and then migrate through a dense extracellular matrix (ECM) to establish productive contact with malignant cells. Each of these steps is substantially hindered within the TME. These physical barriers and representative strategies to overcome them are summarized in Figure 1.

### 3.1. Aberrant vasculature

Tumor blood vessels are structurally and functionally aberrant. They typically exhibit disorganized architecture, heterogeneous perfusion, regional hypoxia, and compromised endothelial integrity. Persistent pro-angiogenic signaling, especially via vascular endothelial growth factor (VEGF)-dependent pathways, can suppress endothelial adhesion molecules such as intercellular adhesion molecule 1 (ICAM1) and vascular cell adhesion molecule 1 (VCAM1). This reduces immune cell adhesion and transendothelial extravasation 55-57. In addition, tumor endothelial cells undergo functional reprogramming. Their inflammatory responsiveness is dampened, and their capacity to support leukocyte transendothelial migration declines. By releasing immunomodulatory mediators, including IL-6, prostaglandin E2 (PGE2), and IL-10, endothelial cells can further reinforce an immunosuppressive vascular niche 55,58-60.

### 3.2. Dense extracellular matrix (ECM)

Once CAR T cells extravasate from the vasculature, they encounter a second bottleneck: migration through a highly fibrotic and disorganized ECM, a hallmark of many solid tumors 61. Dense collagen networks impose direct spatial constraints on T cell motility, particularly when collagen pore size falls below the threshold required for effective interstitial trafficking 62. ECM remodeling also generates functional stress. Increased matrix stiffness and mechanical forces can directly promote T cell dysfunction. In parallel, mechanical compression of blood vessels worsens hypoxia and metabolic stress, further weakening CAR T cell activation and effector capacity 63. Moreover, ECM reorganization can disrupt chemokine gradients and bias tumor-associated macrophages toward immunosuppressive states, together reinforcing immune exclusion 64-67.

Vascular dysfunction and ECM remodeling therefore form an integrated physical barrier that limits CAR T cell delivery, intratumoral infiltration, and productive engagement with tumor cells. This framework has motivated strategies that target vascular and stromal components to improve the spatial accessibility of CAR T cells. At the vascular level, normalization approaches can restore perfusion, alleviate hypoxia, and enhance endothelial inflammatory competence, thereby facilitating extravasation of both endogenous T cells and CAR T cells 68,69. At the stromal level, reducing matrix density or stiffness, locally remodeling ECM architecture, or targeting ECM-producing cells such as cancer-associated fibroblasts can relieve migratory constraints and improve intratumoral CAR T cell distribution 70-72. Overall, overcoming these physical barriers is a prerequisite for achieving robust CAR T cell activity in solid tumors.

### 3.3. Overcoming physical barriers to enhance CAR T cell therapy in lung cancer

In lung cancer, the physical constraints on CAR T cells extend beyond stromal density. They also reflect the compartmentalized airway and alveolar architecture of the lung and its spatially organized immune microenvironments 73. Tumor-driven stromal remodeling further amplifies these spatial restrictions 74. Abundant and functionally heterogeneous cancer-associated fibroblasts (CAFs) in lung lesions can initiate matrix-regulatory and immunomodulatory programs that spatially segregate immune cells from tumor nests. Rather than acting as a uniform population, CAFs comprise multiple functional states with distinct effects on immune localization and regulation. Myofibroblastic CAFs (myCAFs), which are commonly associated with contractile phenotypes and ECM remodeling, may reinforce physical exclusion by promoting collagen deposition, matrix alignment, and stromal stiffening, thereby limiting interstitial migration of T cells and potentially CAR T cells into the tumor parenchyma. In contrast, inflammatory CAFs (iCAFs) are characterized by programs rich in cytokines and chemokines, including IL-6, LIF, CXCL12, and related soluble mediators, which can sustain immune exclusion, recruit suppressive immune populations, and reshape local chemokine gradients. These stromal programs are particularly relevant to adoptive T cell therapy, because even functionally competent T cells may fail to exert effective antitumor activity if they are retained within CAF-rich stromal regions and unable to contact malignant cells 75-77.

Several localized disruption approaches can transiently loosen this compact architecture and recondition the microenvironment, thereby creating a permissive “window” for CAR T cell infiltration and effector activity. In models relevant to lung cancer, radiotherapy, microwave ablation, and nanozyme ablation have each shown synergistic antitumor effects when combined with CAR T cell therapy 78-80. For example, nanozyme ablation followed by infusion of CAR T cells targeting B7-H3 reduced tumor compactness and improved tumor accessibility, resulting in enhanced cytotoxic activity 79. Similarly, microwave ablation combined with AXL-targeted CAR T cells achieved superior tumor control compared with CAR T cell therapy alone 78. In SCLC, chemotherapy may further potentiate CAR T cell efficacy not only by reducing tumor burden, but also by indirectly improving conditions for infiltration and expansion. Proposed mechanisms include relaxation of stromal constraints, increased chemokine expression, and reduction of immunosuppressive cell populations 81.

Beyond direct physical disruption of tumor structure, emerging work has begun to target vascular and perivascular stromal components to improve tissue entry and intratumoral distribution of CAR T cells. Targeting CD248, which is highly expressed by tumor-associated pericytes and perivascular CAFs, has been reported to remodel the aberrant interface between vasculature and stroma and markedly enhance CAR T cell infiltration and antitumor efficacy in lung cancer models 82. Collectively, these findings support direct intervention in tumor vasculature and its supporting stromal scaffold as a promising route to relieve physical constraints and improve therapeutic outcomes.

Overall, physical barriers in lung cancer should not be viewed as static obstacles. Instead, they represent modifiable features that can be strategically manipulated through localized interventions or strategies informed by organ context, thereby increasing opportunities for CAR T cell entry into tumor sites and strengthening antitumor activity.

## 4. Cellular barriers in the TME

Compared with the initial restriction imposed by physical barriers on the spatial accessibility of CAR T cells, cellular barriers are driven by a dynamic network of immunosuppressive populations that directly undermine CAR T cell expansion, survival, and effector function. In lung cancer, this cellular layer of resistance is often more central and difficult to reverse. It also acts in concert with physical, biochemical, and metabolic barriers to further constrain the therapeutic potential of CAR T cell therapy. Regulatory T cells (Tregs), myeloid-derived suppressor cells (MDSCs), and tumor-associated macrophages (TAMs) are frequently enriched in the TME. These populations often outnumber tumor-infiltrating CAR T cells and are closely associated with poor responses to multiple immunotherapies, including CAR T cell therapy 83-86. Sustained gradients of chemokines (for example, CCL2-CCR2, CXCL8/IL-8-CXCR1/2, CXCL12-CXCR4, and CCL22-CCR4) and cytokines (for example, TGF-β, IL-10, IL-6, GM-CSF/G-CSF, and CSF1 (M-CSF)) continuously recruit these suppressive cells into tumors. They also drive highly dynamic phenotypic and functional remodeling during tumor progression and therapeutic pressure 87.

Despite distinct origins and phenotypic diversity, Tregs, MDSCs, and TAMs converge on overlapping suppressive programs. They secrete inhibitory cytokines such as IL-10 and TGF-β, engage checkpoint pathways involving PD-1, PD-L1, and CTLA-4, and compete metabolically with effector T cells. Together, these mechanisms erode CAR T cell function and *in vivo* persistence 83-85.

Importantly, checkpoint signaling in this context should not be viewed as an isolated biochemical pathway. Suppressive cellular populations can reinforce PD-1/PD-L1-dependent inhibition by expressing checkpoint ligands, inducing PD-L1 expression on tumor or stromal cells, and sustaining cytokine programs that increase the activation threshold of CAR T cells. Thus, checkpoint-mediated inhibition represents a functional interface between cellular and biochemical barriers in the lung cancer TME. In addition, TAMs can exacerbate immune exclusion by promoting aberrant angiogenesis and interacting with stromal components, further restricting the intratumoral spatial distribution of CAR T cells 88. On this basis, we summarize representative interventions targeting Tregs, MDSCs, and TAMs across four outcome dimensions relevant to CAR T cell therapy: tumor infiltration, *in vivo* expansion, persistence, and exhaustion (**Table 2**).

### 4.1. Regulatory T cells (Tregs)

Among the suppressive immune populations in the TME, Tregs are a prominent barrier to effective CAR T cell responses, owing to both their numerical advantage and potent inhibitory capacity. Enrichment of intratumoral Tregs is consistently associated with reduced efficacy of immune checkpoint blockade, bispecific T cell engagers, and CAR T cell therapy 86,120-122. In the context of CAR T cell therapy, Treg-mediated suppression can arise through multiple routes. It may reflect ongoing recruitment and expansion of endogenous tumor-resident Tregs. It may also be introduced during manufacturing, through inadvertent CAR transduction of Tregs within the starting T cell population. Clinical evidence indicates that a higher proportion of CAR^+^ Tregs in the infusion product is significantly associated with lack of response to CAR T cell therapy targeting CD19. Notably, CAR design features and specific manufacturing workflows can influence the frequency of CAR Tregs, providing a rationale for process optimization to mitigate this risk 123,124. Treg accumulation within tumors is shaped by defined chemokine axes, including trafficking through CCR4 and CCR8. Once established in the TME, Tregs can preferentially survive and expand under suppressive cytokine cues (notably TGF-β and IL-10) and harsh metabolic conditions characterized by hypoxia, acidosis, and nutrient scarcity. These factors together sustain Treg suppressive function and reinforce a stable cellular barrier against CAR T cell activity 125,126.

### 4.2. Myeloid-derived suppressor cells (MDSCs)

MDSCs constrain CAR T cell expansion and durable antitumor activity through highly plastic and pleiotropic suppressive programs. Increased MDSC abundance in the TME or peripheral blood is closely associated with adverse outcomes following CAR T cell therapy 127,128. Mechanistically, MDSCs are preferentially recruited to tumors through key chemokine pathways, including CXCR2, CCR2, and CCR5 129. Once established, they undermine CAR T cell function and survival via multiple parallel mechanisms. These include metabolic competition, reactive oxygen species (ROS) production, secretion of inhibitory cytokines such as IL-10 and TGF-β, and depletion of immunologically critical amino acids, including arginine and tryptophan 130.

Importantly, suppression mediated by MDSCs is often reversible. Pharmacologic or immune strategies that reduce MDSC abundance or directly disrupt their suppressive programs can restore CAR T cell proliferation, effector function, and persistence, thereby improving overall antitumor efficacy 106,131,132. These features position MDSCs as a highly actionable partner for combination strategies designed to enhance CAR T cell therapy in solid tumors.

### 4.3. Tumor-associated macrophages (TAMs)

By contrast, TAMs represent the numerically dominant suppressive compartment in many solid tumors and constitute a central component of cellular barriers to CAR T cell therapy. TAM recruitment is driven largely by the CCL2-CCR2 axis, and enrichment of M2-like states is closely linked to poor prognosis across multiple solid tumor types 133,134. TAMs suppress CAR T cell activity through both direct and indirect mechanisms. Directly, they secrete inhibitory cytokines such as IL-10 and TGF-β and express checkpoint ligands including PD-L1, thereby linking suppression by macrophages to inhibition of CAR T cell activation through the PD-1/PD-L1 axis. Indirectly, they promote aberrant angiogenesis and stromal remodeling, which further restricts CAR T cell infiltration.

Accordingly, targeting TAMs has become an important strategy to improve CAR T cell efficacy. Reprogramming TAMs toward pro-inflammatory, M1-like phenotypes using CD40 agonists, TLR ligands, or CSF-1R inhibitors can be more effective than indiscriminate depletion in restoring T cell function and reshaping the immune microenvironment 119,135-137. In addition, phagocytosis of tumor material by TAMs has been proposed as a mechanism contributing to resistance to CAR T cell therapy, motivating engineering approaches to mitigate macrophage-driven clearance or rewire myeloid checkpoints 118. For example, armored CAR T cells engineered to secrete factors that reprogram TAMs (such as anti-IL-10 antibodies or GM-CSF antagonists), or CAR T cells modified to resist TAM-mediated phagocytic mechanisms, may offer complementary routes for combination therapy 118.

Overall, Tregs, MDSCs, and TAMs form a highly dynamic and mutually reinforcing immunosuppressive network. This network cooperates with physical and biochemical barriers to systematically restrict CAR T cell infiltration, survival, and effector function.

### 4.4. Overcoming cellular barriers to enhance CAR T therapy in lung cancer

In lung cancer, immunosuppressive cellular programs can emerge early in disease and are frequently reshaped by prior lines of therapy, including chemotherapy, radiotherapy, and immune checkpoint blockade 138. As a result, before infusion, patients enrolled in CAR T cell trials may already have a TME enriched in suppressive myeloid populations and sustained inhibitory checkpoint signaling. This creates an unfavorable baseline for subsequent cellular therapy 138-140. To address this challenge, the field has moved beyond simply “depleting” suppressor cells. Current approaches increasingly combine active rewiring of the TME with engineering of more resilient CAR T cell products that can adapt to context. A more direct strategy is to convert suppressive myeloid cells into therapeutic entry points. Recent work showed that IL-12-armored CAR T cells targeting FOLR2+ or TREM2+ TAMs depleted suppressive macrophages, expanded CXCL9+ immunostimulatory macrophages, and activated endogenous CD8+ T cells. Importantly, IL-12-armored anti-TREM2 CAR T cells reduced tumor burden and prolonged survival in a metastatic lung cancer model, supporting TAM-directed CAR T engineering as a strategy to convert myeloid barriers into drivers of antitumor immunity 141.

Another strategy is to indirectly reprogram suppressive myeloid circuits through regimen design. Adding oxaliplatin to lymphodepletion has been reported to activate TAMs and induce pro-inflammatory chemokines, thereby reshaping the TME and promoting recruitment and activity of ROR1-targeted CAR T cells in lung tumor models 31. This concept is further supported in SCLC, which is often characterized by low T cell infiltration alongside amplified myeloid immunosuppression. In this setting, DLL3-targeted CAR T cells engineered to secrete a CD47-blocking protein can disrupt CD47-SIRPα “don't eat me” signaling. This shifts TAM function from immunosuppression toward tumor phagocytosis, effectively converting an immune barrier into an antitumor effector mechanism 142. In addition, lung cancer models suggest that CAR T cell derived exosomes (CAR-Exos) can mediate direct tumor cell killing and substantially increase intratumoral CD8⁺ T cell infiltration, partially offsetting the numerical dominance of suppressive immune populations 143. Together, these findings highlight the importance of restructuring suppressive cellular networks in lung cancer.

Nanotechnology-enabled modulation of the TME also shows translational potential. In lung cancer models, PD-L1-targeted nanovesicles delivering a STING agonist can simultaneously reprogram inflammatory signaling and checkpoint pathways, reduce myeloid immunosuppression, promote CAR T cell infiltration, and alleviate exhaustion 144. Immunomodulatory nanoplatforms, exemplified by CuS photothermal nanoparticles, can selectively reduce MDSCs while enhancing CD8⁺ and CD4⁺ T cell infiltration and limiting regulatory Treg enrichment in lung tumor models 145. Although these platforms have not yet been broadly integrated with CAR T cell therapy, they support a clinically tractable principle: reshaping myeloid suppression while improving T cell access may provide effective combination strategies for lung cancer.

Building on these concepts, integrated delivery systems that directly couple CAR T cell therapy with microenvironmental interventions are beginning to emerge. For example, mesenchymal stem cell-mediated delivery of an oncolytic adenovirus encoding IL-12 and a PD-L1 blocker substantially enhanced the antitumor efficacy of HER2-targeted CAR T cells in preclinical models 146. By amplifying pro-inflammatory cues while relieving checkpoint inhibition, such combinatorial delivery strategies can partially reverse tolerance maintained by multiple suppressive immune compartments.

Taken together, cellular resistance in lung cancer is not driven by any single immunosuppressive subset. Rather, it reflects the combined influence of prior treatment history, checkpoint signaling, and the functional states of myeloid cells. To overcome this durable and evolving network, CAR T cell therapy will likely need to be integrated with TME-modulating strategies. Such coordinated approaches may be required to achieve more robust and sustained therapeutic effects in this immunosuppression-dominant solid tumor setting.

## 5. Biochemical and metabolic barriers in the TME

Beyond physical and cellular constraints, CAR T cells that reach solid tumors are exposed to a profoundly suppressive biochemical and metabolic milieu. This environment is shaped by immunosuppressive cytokines, persistently engaged immune checkpoint signaling, dysregulated chemotactic gradients, and an extremely nutrient-poor and hostile metabolic landscape. Together, these factors undermine CAR T cell activation, persistence, and effector function across multiple levels, spanning signal transduction, cellular positioning, and energy supply. Ultimately, they promote dysfunction and exhaustion 72.

### 5.1. Immunosuppressive soluble factors

The TME is often enriched in immunosuppressive cytokines, with TGF-β and IL-10 as prototypical examples. These factors are produced by tumor cells, TAMs, Tregs, and other stromal components 126,147. They can dampen CAR-associated signaling, reduce effector cytokine production, and induce transcriptional programs linked to T cell dysfunction, thereby weakening the antitumor activity of CAR T cells.

### 5.2. Immune checkpoint signaling

As discussed above, immune checkpoint signaling is closely reinforced by suppressive cellular populations in the lung cancer TME. At the same time, immune checkpoint pathways also represent a major biochemical mechanism that directly restrains CAR T cell activation and effector function. Sustained activation of the PD-1/PD-L1 and CTLA-4 axes across tumor cells, stromal cells, and suppressive immune populations can markedly impair CAR T cell activity 148. PD-1 signaling limits T cell activation, proliferation, and survival in part by suppressing CD28-dependent PI3K-Akt signaling 149,150. In parallel, CTLA-4 competitively antagonizes CD28-CD80/CD86 costimulation, deepening hyporesponsiveness and promoting functional exhaustion 151.

### 5.3. Chemokine dysregulation

Chemokine dysregulation represents another major biochemical barrier. Many solid tumors exhibit a “chemokine desert” phenotype. They lack T helper 1 (Th1)-type chemokines that recruit cytotoxic lymphocytes, such as CXCL9 and CXCL10. Restoring these CXCR3 ligands has been shown to improve T cell infiltration and enhance responses to immunotherapy 152,153. In parallel, tumors often overexpress chemokines such as CCL2, CCL5 and CXCL12. These signals preferentially recruit suppressive populations, including Tregs and MDSCs, into the tumor microenvironment. This skewed recruitment impairs CAR T cell homing and spatial distribution. It also reinforces immune suppression, strengthens immune exclusion, and in some settings helps sustain hypoxia 154-156.

### 5.4. Metabolic competition

Aberrant tumor metabolism constitutes the deepest layer of metabolic constraint. Hypoxia, nutrient deprivation (notably glucose and glutamine), and extracellular acidification together create a hostile metabolic niche. In this setting, CAR T cells face an energy crisis and direct metabolic competition with tumor cells 157,158. Hypoxia activates hypoxia-inducible factors (HIF-1α and HIF-2α). This can upregulate inhibitory molecules such as PD-L1, VISTA and CD47. It may also promote immune evasion by triggering autophagy and inducing tumor expression of HLA-G and HLA-E 159-161. In addition, metabolites enriched in tumors and stromal compartments, including prostaglandin E2 (PGE2), adenosine and ROS, directly suppress T cell metabolic programs and effector function through receptors such as A2AR. These cues accelerate CAR T cell dysfunction and exhaustion 162,163.

**Figure 2:**
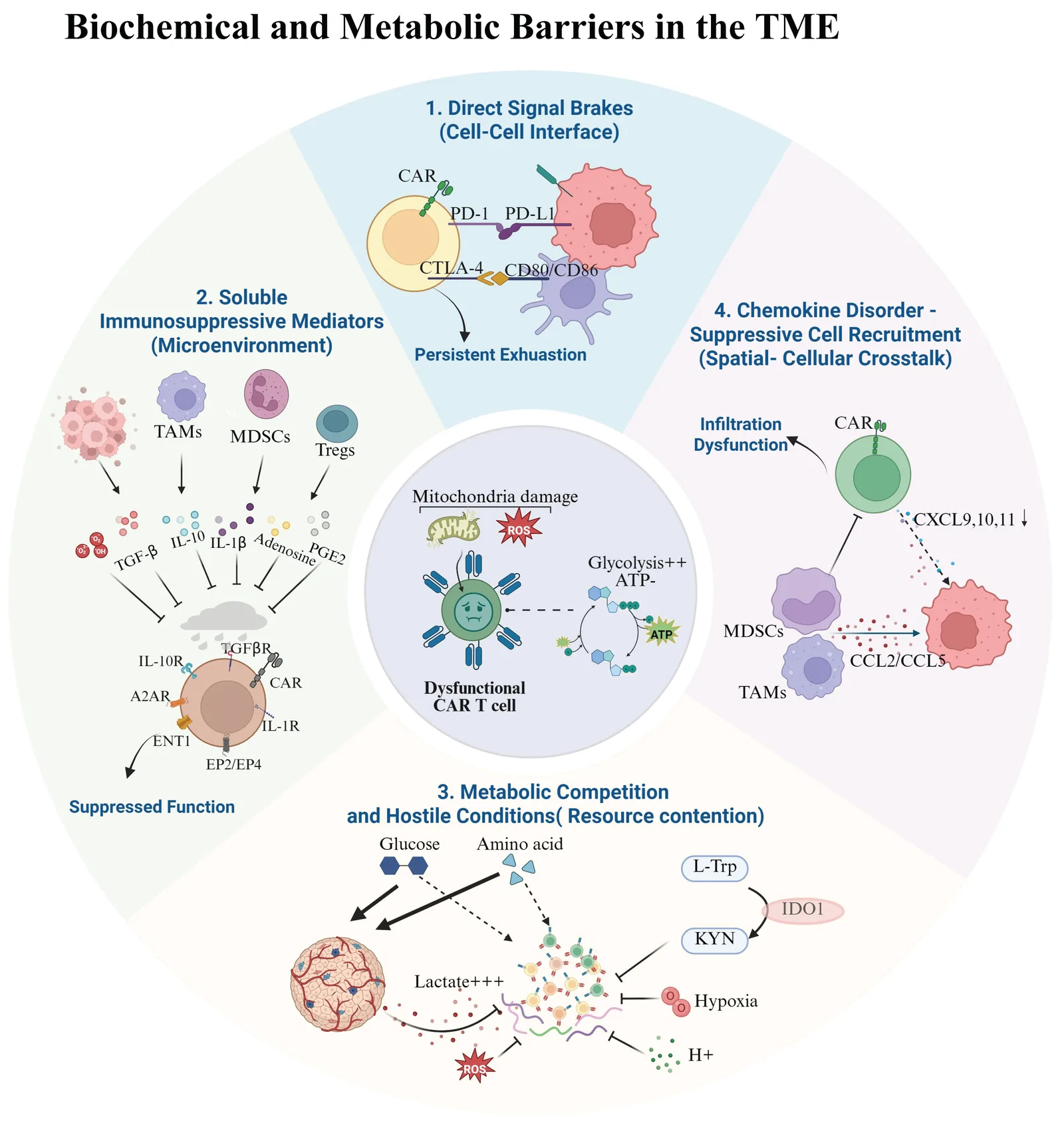
** Biochemical and metabolic barriers driving CAR T cell dysfunction in the TME.** CAR T cells entering tumors encounter layered non-physical suppression barriers. These include checkpoint-mediated signal brakes at the cell-cell interface, soluble immunosuppressive mediators, metabolic competition and hostile conditions (hypoxia, nutrient depletion, lactate and adenosine), and chemokine dysregulation that impairs homing while recruiting suppressive cells. Together, these cues converge to promote CAR T cell dysfunction and persistent exhaustion (Created with BioRender.com).

Under sustained biochemical and metabolic pressure, CAR T cells progressively acquire an intrinsic exhaustion program. This is characterized by reduced proliferative capacity, weakened cytotoxicity, diminished cytokine production, and persistent upregulation of inhibitory receptors 164,165. Collectively, biochemical and metabolic barriers form a critical “non-cellular” layer of resistance after CAR T cells enter tumors. By continuously rewiring signaling, metabolic fitness, and transcriptional state, they impose a system-level constraint on CAR T cell efficacy in solid tumors (Figure 2) 72.

### 5.5. Overcoming biochemical and metabolic barriers to enhance CAR T cell therapy in lung cancer

The PD-1/PD-L1 axis has broad relevance to lung cancer biology and treatment. Immune checkpoint inhibitors have become a cornerstone of therapy for non-small cell lung cancer (NSCLC), underscoring the functional importance of this pathway within the lung cancer tumor microenvironment 166. Preclinical studies further show that dampening PD-1/PD-L1 signaling can restore and amplify CAR T cell activity in lung cancer models. This can be achieved either by combining CAR T cells with systemic PD-1/PD-L1 blockade or by engineering CAR T cells to resist checkpoint inhibition, for example through PD-1 gene knockout or expression of a dominant-negative PD-1 receptor 15.

To address CAR T cell recruitment and positioning failures driven by dysregulated chemokine gradients in lung tumors, current strategies emphasize guided homing and local rewiring. Engineering CAR T cells to co-express selected chemokine receptors (such as CXCR5 or CCR6) enables them to actively respond to cognate ligands enriched at tumor sites, thereby improving trafficking to lung tumor regions 14,167. These findings support the concept that reprogramming chemokine axes can partially relieve biochemical barriers in lung cancer. In addition, in SCLC models, equipping CAR T cells with the capacity to secrete defined chemokines or cytokines (for example IL-7, IL-18, or CCL19) has been reported to enhance antitumor activity and promote intratumoral immune remodeling, outperforming conventional CAR T cells 41,168.

As noted above, many patients with lung cancer have already received multiple lines of systemic therapy, including chemotherapy, before CAR T cell treatment. Chemotherapy not only reduces tumor burden, but can also indirectly shape CAR T cell infiltration and function by rewiring local cytokine profiles, chemokine networks, and metabolic stress. Certain agents trigger damage-associated programs and increase chemokine expression, thereby creating more permissive conditions for CAR T cells. For example, docetaxel has been reported to upregulate CXCL11 and promote HMGB1 release, which enhanced infiltration of HER2-targeted CAR T cells and improved therapeutic outcomes in lung cancer models 34. Similarly, regimens based on oxaliplatin can increase tumor sensitivity to PD-L1 blockade and synergistically augment CAR T cell antitumor activity 31. In addition, a preclinical NSCLC model showed that intratumoral delivery of engineered dendritic cells that continuously secrete CXCL9 and CXCL10 enhanced T cell infiltration and functional activation and suppressed tumor growth 169. Together, these findings suggest that rationally leveraging the immunomodulatory effects of chemotherapy and integrating them into CAR T cell preconditioning regimens is a practical route to improve efficacy using clinically available tools.

On the metabolic side, directly targeting dominant suppressive signals generated by tumor metabolism offers a rapid entry point to restore CAR T cell function. Adenosine is produced at high levels by tumor and stromal cells through the CD39/CD73 enzymatic axis and suppresses T cell activation and proliferation through adenosine A2A receptor (A2AR) signaling. In lung cancer models, knockout of A2AR in CAR T cells using CRISPR/Cas9 significantly enhanced antitumor activity, highlighting the potential of targeting the adenosine pathway to relieve metabolic suppression 170.

In addition, metabolic programming during *ex vivo* manufacturing can be used to precondition CAR T cells for improved fitness and persistence after infusion. Distinct T cell subsets rely on different metabolic circuits. Highly glycolytic effector T cells are more prone to dysfunction and exhaustion, whereas memory populations, such as memory stem T cells and central memory T cells, preferentially use oxidative phosphorylation and fatty acid oxidation and exhibit superior long-term survival and proliferative capacity. 171. Accordingly, limiting excessive glycolysis while preserving mitochondrial metabolism during manufacturing may reduce the risk of intrinsic exhaustion. Supplementation with cytokines such as IL-7, IL-15, IL-21, and IL-10, as well as short-chain fatty acids or alternative carbon sources, has been reported to promote memory-like CAR T cell states and enhance antitumor function 172-176. Notably, combined IL-7 and IL-15 conditioning produced a clear sensitizing effect in an EGFR-targeted CAR T cell lung cancer model 171.

Building on the intertwined nature of metabolic suppression and chemotactic dysfunction, “co-engineering” strategies based on intelligent nanodelivery systems are advancing rapidly. For example, a tumor microenvironment-responsive nanoimmunomodulator, FMANAC, has been designed to release the chemokine CCL5 under acidic conditions to improve T cell recruitment, while simultaneously releasing the indoleamine 2,3-dioxygenase (IDO) inhibitor NLG919 to relieve tryptophan metabolism-driven immunosuppression. When combined with CD276-targeted CAR T cells, this approach significantly enhanced antitumor efficacy in the H460 lung cancer model 177. Similarly, programmable anti-PD-L1 nanovesicles have been used to deliver a glutamine antagonist in a targeted manner. This strategy suppresses tumor metabolism while concurrently blocking checkpoint signaling, thereby improving CAR T cell infiltration and promoting durable antitumor activity in an orthotopic lung cancer model 178.

More broadly, the lung cancer microenvironment is typified by hypoxia, competitive glucose depletion, and lactate accumulation, which together create a deep metabolic barrier that restrains T cell function. In response, multiple engineering approaches have been proposed to improve the metabolic adaptability of CAR T cells. One direction is intrinsic metabolic rewiring, enabling CAR T cells to function under resource-limited conditions. Examples include hypoxia-responsive CAR T cells or strategies that enhance glucose uptake to sustain proliferation and effector activity in metabolically hostile niches 179,180. A complementary direction is extrinsic metabolic remodeling, in which biomimetic or catalytic systems are deployed to improve the local metabolic ecosystem encountered by CAR T cells. For instance, a nanocatalytic platform camouflaged with CAR T cell membranes has been reported to inhibit tumor glycolysis and reduce lactate levels, indirectly alleviating chemical stress within the tumor milieu 181. Although these approaches have not yet been systematically validated in lung cancer models, they target metabolic features that are highly prevalent in lung tumors, supporting clear translational potential. Collectively, these interventions form a multidimensional toolkit to overcome chemotactic and metabolic suppression and reinvigorate CAR T cell activity in lung cancer.

## 6. Combination strategies to enhance CAR T therapy in lung cancer

**Figure 3:**
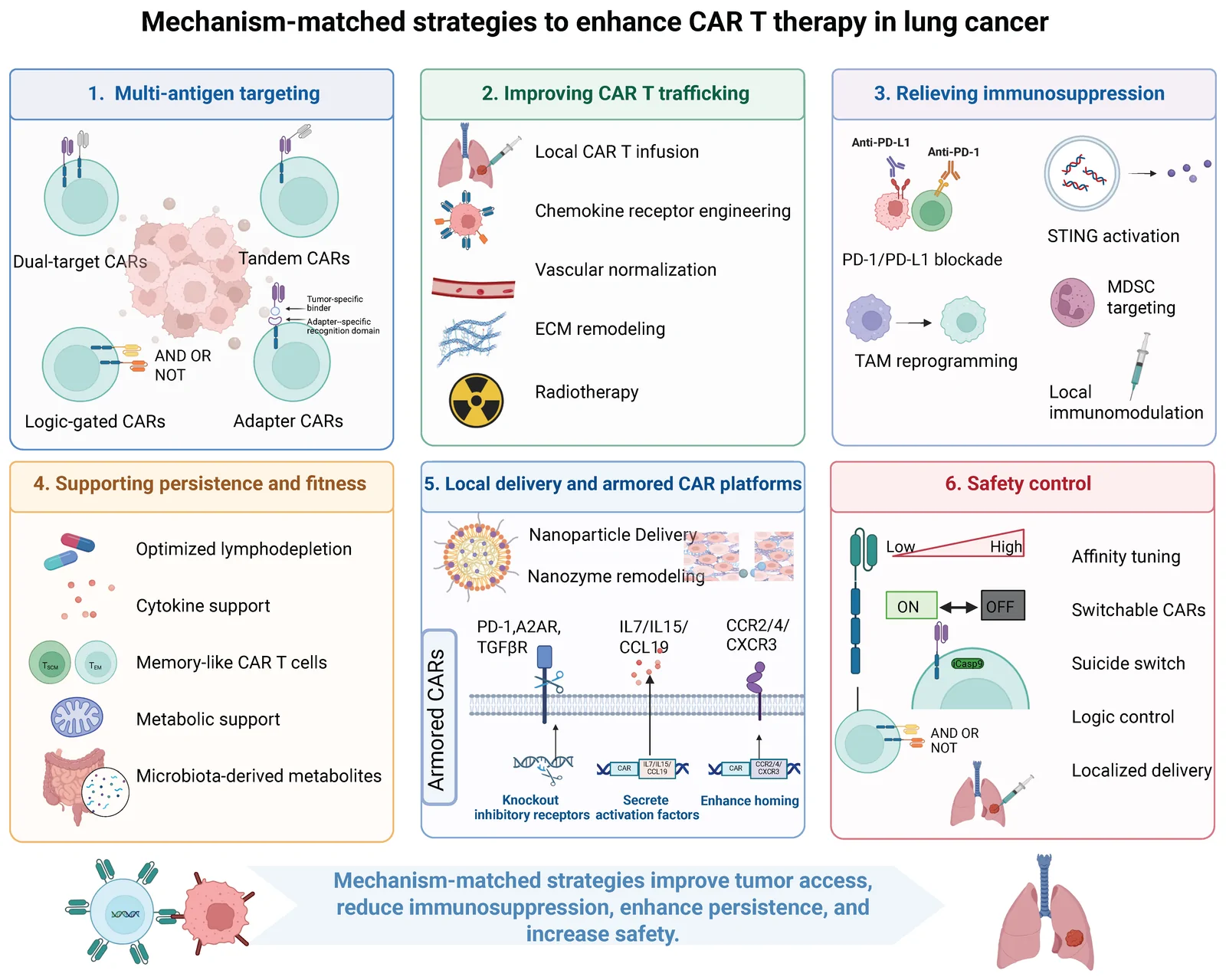
** Mechanism-matched strategies to enhance CAR T therapy in lung cancer.** The schematic summarizes representative strategies to improve CAR T therapy in lung cancer, including multi-antigen targeting, enhanced CAR T trafficking, relief of immunosuppression, persistence and fitness support, local delivery and engineered CAR platforms, and safety-control approaches. These barrier-matched strategies aim to improve tumor access, reduce TME-mediated suppression, enhance CAR T cell persistence, and increase therapeutic safety (Created with BioRender.com).

Given these multilayered barriers, the goal of combination strategies in lung cancer CAR T therapy is not simply to add more treatments, but to use TME priming or context-matched interventions to convert antigen recognition into more durable intratumoral effector function (Figure 3).

Current studies in lung cancer suggest that optimization of chemotherapy or lymphodepletion, local ablation, radiotherapy, nanodelivery, and local immunomodulatory platforms can all serve as approaches for TME priming. Chemotherapy and lymphodepletion not only reduce tumor burden, but may also create a window for CAR T cell recruitment and expansion by inducing pro-inflammatory chemokines, altering myeloid cell states, and enhancing antigen release. For example, immunogenic chemotherapy has been shown to enhance CAR T cell recruitment to lung tumors and to further improve antitumor activity when combined with checkpoint blockade 31. Local ablation, radiotherapy, and structural remodeling by nanozymes may transiently loosen dense tumor architecture and thereby improve CAR T cell access to the tumor parenchyma. Studies of inhaled PD-L1-targeted nanovesicles loaded with a STING agonist, nanozyme-mediated TME remodeling, and armed oncolytic viruses delivering immunomodulatory payloads further support local immune modulation as a promising strategy to reshape the lung cancer TME before or during CAR T therapy 144,182.

Checkpoint blockade is another important combination strategy, but its role should be interpreted in the context of antigen presentation status and the immune background of the TME. The limited benefit of PD-L1 blockade in SCLC suggests that releasing PD-1/PD-L1 inhibition alone is often insufficient to restore effective antitumor immunity. Defective MHC-I antigen presentation and an immune-protective TME may still restrict cytotoxic T cell recognition and tumor cell clearance 183. Therefore, checkpoint blockade may be more effective when paired with strategies that improve tumor accessibility, broaden antigen coverage, or remodel myeloid suppression.

Beyond studies specific to lung cancer, several combination approaches reported in other solid tumors may also provide useful directions for CAR T therapy. Anti-angiogenic therapy may promote vascular normalization, improve perfusion, and increase lymphocyte entry through VEGF blockade. Although direct evidence in lung cancer CAR T therapy remains limited, related CAR T models support its feasibility in improving cell delivery and antitumor efficacy. Cancer vaccines may also serve as an adjunctive module. For example, a CLDN6 RNA vaccine combined with CLDN6 CAR T cells showed preliminary signals of disease control in CLDN6-positive relapsed or refractory solid tumors, suggesting that vaccines may improve persistence by enhancing antigen stimulation or supporting CAR T cell expansion 184. In addition, microbiota-derived short-chain fatty acids have been reported to promote CAR T cell persistence and antitumor activity, indicating that metabolic support may represent another avenue for future optimization 174.

Future studies should further refine combination strategies based on dominant resistance mechanisms. Tumors dominated by poor infiltration and spatial exclusion may be better suited to combinations with vascular or stromal remodeling, local ablation, or radiotherapy. Tumors characterized mainly by checkpoint signaling and myeloid suppression may benefit more from checkpoint blockade, STING activation, or TAM/MDSC reprogramming. In settings where limited persistence or functional exhaustion predominates, more appropriate conditioning regimens, metabolic support, or maintenance strategies may be required (Figure 3). Future studies should define optimal treatment sequencing, delivery routes, toxicity control, patient selection, and monitorable TME biomarkers to ensure that increasingly complex combination strategies translate into durable clinical benefit.

## 7. Conclusion and Future Perspectives

The success of CAR T cell therapy in hematologic malignancies has demonstrated its strong therapeutic potential, but its translation into solid tumors such as lung cancer remains substantially limited. Current development of CAR T therapy in lung cancer has covered multiple candidate targets and shown early feasibility; however, stable, durable, and reproducible clinical benefit has not yet been established. This gap indicates that the central challenge in lung cancer CAR T therapy is not simply a lack of ideal targets, but the failure to achieve an effective match among target recognition, therapeutic window, tumor accessibility, TME suppression, and CAR T cell persistence.

Among these constraints, the TME is a critical arena that determines whether CAR T activity can be converted into therapeutic efficacy in lung cancer. Aberrant vasculature, dense stroma, immunosuppressive cellular networks, checkpoint signaling, chemokine dysregulation, and metabolic stress do not operate in isolation. Instead, they form a dynamic and mutually reinforcing resistance system. Within this system, CAR T cells may recognize tumor antigens but still progressively lose effector activity because of poor tumor entry, functional suppression, failed metabolic adaptation, or persistent antigen stimulation. Therefore, understanding and reshaping the lung cancer TME should not be viewed as an auxiliary component of CAR T therapy, but as a central strategy for improving its clinical translation.

Future development of CAR T therapy in lung cancer should move beyond simply enhancing CAR T cell cytotoxicity toward a more integrated design based on defined mechanisms. Safer and more adaptable CAR T products should be combined with rationally selected TME-modulating interventions and combination strategies, and further matched to each patient's antigen status, degree of immune exclusion, stromal architecture, and metabolic pressure. Future studies should also define optimal treatment sequencing, delivery routes, toxicity management, and monitorable TME biomarkers. Ultimately, whether CAR T therapy can achieve consistent benefit in lung cancer will depend on its ability to continuously convert antigen recognition into effective intratumoral tumor killing within a complex, dynamic, and immunosuppressive TME.

## Figures and Tables

**Table 1:** Representative CAR T targets, translational evidence, and key challenges in lung cancer

Target	Lung cancer type	Biological characteristics	Representative preclinical / clinical evidence	Clinical trial examples	Key translational concerns	References
EGFR	EGFR-positive advanced/refractory NSCLC	Frequently altered or overexpressed in NSCLC and involved in tumor proliferation, invasion, and survival.	EGFR CAR T cells showed EGFR-specific cytotoxicity and early clinical feasibility. PiggyBac-engineered EGFR CAR T was reported to be feasible and safe, while CXCR5-modified EGFR CAR T was designed to improve trafficking through the CXCR5-CXCL13 axis.	NCT01869166 NCT03182816 NCT04153799 NCT05060796 NCT06682793 NCT05341492	Normal epithelial expression may increase on-target/off-tumor risk; efficacy remains modest; tumor infiltration and TME suppression remain limiting factors.	12-16
MSLN	NSCLC, lung adenocarcinoma, and malignant pleural disease	Overexpressed in NSCLC and involved in tumor proliferation, invasion, and metastasis, with restricted expression in most normal tissues.	MSLN CAR T cells have shown early clinical feasibility. Low-dose MSLN CAR T was reported to be safe and feasible, whereas high-dose infusion caused severe pulmonary toxicity. Safety-enhanced designs, including iCasp9-MSLN CAR T and checkpoint nanobody-secreting MSLN CAR T, have been explored.	NCT03054298 NCT02414269 NCT04489862 NCT06248697 NCT01583686 NCT06051695 NCT06885697	Potential pulmonary toxicity; low-level expression in benign lung tissues; need for dose optimization, safety switches, and regional delivery strategies.	17-19
MUC1 / TnMUC1	Advanced NSCLC and epithelial solid tumors	Overexpressed and aberrantly glycosylated in NSCLC and involved in tumor invasion, stemness, and therapy resistance.	MUC1 CAR T cells showed antitumor activity in preclinical solid-tumor models. In NSCLC, MUC1/PSCA dual targeting and MUC1 CAR T with PD-1 disruption have been explored to enhance efficacy; stable disease was reported in a subset of advanced NSCLC patients receiving MUC1 CAR T with PD-1 disruption.	NCT02587689 NCT03356808 NCT04025216 NCT03525782	Limited objective responses; antigen heterogeneity; unclear durability; may require multi-target, checkpoint-resistant, or TME-primed strategies.	20-23
PSCA	NSCLC and multi-antigen lung cancer CAR T strategies	Overexpressed in NSCLC and involved in lung cancer cell growth, supporting its potential as a surface target.	PSCA CAR T showed preclinical antitumor activity in lung cancer models. MUC1/PSCA dual targeting enhanced antitumor efficacy compared with single-antigen targeting in NSCLC models.	NCT03198052	Limited lung cancer-specific clinical evidence; antigen distribution and safety require further validation; likely best positioned in multi-target strategies.	20,23,24
CEA	CEA-positive lung cancer and other solid tumors	Overexpressed in lung cancer and involved in tumor cell adhesion, with clinical relevance as a tumor marker.	CEA CAR T has been evaluated in CEA-positive solid tumors, including lung cancer. Early studies suggest feasibility, but lung cancer-specific efficacy remains insufficiently defined.	NCT02349724 NCT04348643 NCT06903117 NCT06992583 NCT06768151 NCT07250386 NCT07179692 NCT06006390 NCT06010862 NCT06126406 NCT06043466	Not lung cancer-specific; heterogeneous expression; limited direct NSCLC outcome data; potential off-tumor toxicity should be considered.	25-27
PD-L1	NSCLC and immunosuppressive lung cancer TME	PD-L1 is a classical checkpoint ligand that suppresses T-cell activation and function and is expressed on tumor, stromal, and immune cells.	PD-L1 CAR T showed preclinical tumor-killing activity in NSCLC models. However, in NCT03330834, only one patient was enrolled and serious CRS occurred after CAR T infusion.	NCT03330834 NCT03060343 NCT06348797 NCT04684459	Not tumor-specific; risk of on-target/off-tumor toxicity and CRS; requires affinity tuning, logic gating, switchable control, or local delivery.	28-30
ROR1	Advanced NSCLC and ROR1-expressing solid tumors	Overexpressed in lung cancer and involved in tumor proliferation, migration, and poor prognosis.	ROR1 CAR T showed activity in preclinical lung cancer models. Combination with oxaliplatin-based lymphodepletion and PD-L1 blockade improved ROR1 CAR T-mediated lung tumor control in preclinical studies.	NCT02706392	Safety and efficacy in NSCLC remain unclear; TME pressure and PD-L1 upregulation may blunt ROR1 CAR T activity; rational combination with checkpoint blockade or TME priming may be needed.	31-33
HER2	HER2-altered or HER2-overexpressing NSCLC	Mutated or overexpressed in a subset of lung cancers and involved in tumor invasion and metastasis.	HER2 CAR T has mainly been studied preclinically in lung cancer. Chemotherapy-mediated recruitment and HMGB1-related modulation have been explored to enhance HER2 CAR T activity in NSCLC models.	NCT01935843 NCT03198052 NCT02713984	Normal tissue expression raises safety concerns; lung cancer-specific clinical data remain limited; careful dose and target-density control are needed.	34-36
B7-H3 / CD276	NSCLC, SCLC, and metastatic lung cancer models	Overexpressed in lung cancer and involved in immune suppression, poor prognosis, and disease progression, with limited expression in most normal tissues.	B7-H3 CAR T showed preclinical cytotoxicity against lung cancer. CCR2b-enhanced B7-H3 CAR T improved trafficking in NSCLC brain metastasis models, and nanozyme-based TME remodeling enhanced B7-H3 CAR T activation and infiltration in NSCLC models.	NCT03198052 NCT04864821 NCT05341492	Heterogeneous expression; TME-mediated suppression; trafficking barriers, especially in metastatic or brain-involved disease; clinical efficacy remains unproven.	37,38
DLL3	SCLC and large-cell neuroendocrine lung carcinoma	Highly expressed in SCLC and neuroendocrine lung cancers with limited normal-tissue expression, supporting its potential as a lineage-associated surface target.	DLL3 CAR T showed preclinical efficacy and safety in SCLC models. DLL3-targeted CAR T trials have recently been initiated in relapsed/refractory SCLC and large-cell neuroendocrine lung carcinoma.	NCT05680922 NCT06384482 NCT06348797 NCT07246304 NCT07249879 NCT07395479	SCLC subtype plasticity and antigen heterogeneity may drive escape; clinical durability and safety remain unproven; may require combination or armored strategies.	39-41
GD2	SCLC, NSCLC, and GD2-expressing lung cancer models	Expressed in a subset of SCLC and NSCLC and involved in tumor proliferation, migration, adhesion, and invasion.	GD2 CAR T showed preclinical activity in orthotopic and metastatic lung cancer models. IL-15-armored GD2 CAR T with an iCasp9 safety switch showed improved expansion, persistence, and antitumor activity, while tazemetostat could upregulate GD2 expression in GD2-low/negative lung cancer cells.	NCT05620342 NCT03356808	Heterogeneous expression; potential neurotoxicity and normal tissue expression; limited lung cancer-specific clinical data; may require antigen-upregulation or safety-switch strategies.	42
MAGE-A1 / MAGE-A4	Antigen-expressing lung cancer and multi-antigen CAR T strategies	Cancer-testis antigens with restricted normal-tissue expression, but generally intracellular rather than surface antigens.	MAGE-family antigens have been included in lung cancer multi-antigen CAR T exploration; however, their intracellular localization makes them more directly relevant to TCR-T strategies than conventional surface-targeted CAR T.	NCT03356808	Intracellular localization limits conventional CAR targeting; requires careful validation of surface accessibility or alternative TCR-based approaches.	43
PTK7	SCLC and PTK7-expressing lung cancer models	Expressed in SCLC and involved in cell polarization, migration, chemotaxis, and tumor progression.	PTK7 CAR T showed preclinical antitumor activity against PTK7-high SCLC cells and prolonged survival in SCLC xenograft models.	No lung cancer-specific clinical trial identified	Target distribution, tumor specificity, safety, and clinical relevance remain insufficiently defined.	44

**Abbreviations:** CAR, chimeric antigen receptor; NSCLC, non-small cell lung cancer; SCLC, small cell lung cancer; TME, tumor microenvironment; EGFR, epidermal growth factor receptor; MSLN, mesothelin; MUC1, mucin 1; PSCA, prostate stem cell antigen; CEA, carcinoembryonic antigen; PD-L1, programmed death-ligand 1; ROR1, receptor tyrosine kinase-like orphan receptor 1; HER2, human epidermal growth factor receptor 2; DLL3, delta-like ligand 3; PTK7, protein tyrosine kinase 7; CRS, cytokine release syndrome.

**Table 2:** Targeting Tregs, MDSCs and TAMs to enhance CAR T cell efficacy

Cell type	CAR T infiltration	CAR T expansion	CAR T persistence	CAR T exhaustion
Tregs	Recruitment blockade: FLX475 (CCR4 antagonist) 89 anti-CCR8 mAb 90	Depletion: anti-CD25 91,92 anti-CTLA-4 93	Reduce IL-2 dependence / bypass IL-2 sink: IL-2 axis-tuned CAR designs (e.g., CD28 endodomain tuning) 94 autocrine cytokine-support frameworks (e.g., IL-7 loop) 95	Disarm Treg program / harden CAR T against suppression: FOXP3-directed approaches 96 IL-2 axis-tuned CAR designs 94
MDSCs	Recruitment blockade: CXCR2/CCR2/CCR5 axis inhibitors or Abs 97-99 CAF-CXCL12 axis disruption 100 HDAC/DNMT inhibitors 101 1,25(OH)₂D₃ 102	Depletion: CD33-directed depletion (gemtuzumab ozogamicin) 103 TRAIL-R2/DR5 agonism (DS-8273a) 104 TKIs (sunitinib/lenvatinib) 105,106	Functional reprogramming: All-trans retinoic acid (ATRA) 107	Blunt suppressive chemistry: Arg1/NOS2 targeting 108 ROS-limiting approaches (triterpenoid) 109 FAO inhibition 110 FATP2 inhibition 111
TAMs	Recruitment blockade: CCL2-CCR2 blockade 112 AHR inhibitors 113	Depletion: CSF1R inhibition 114 FRβ-targeted CAR T 115 CD206/FRβ-targeted BiTEs 116	Prevent CAR T loss / enable co-therapy: CD40 agonists 117 CD47-axis-compatible CAR designs (e.g., epitope-edited CD47 enabling anti-CD47) 118	Reverse macrophage-driven suppression: TLR7/8 agonism 119

## Abbreviations

A2AR: adenosine A2A receptor
CAFs: cancer-associated fibroblasts
CAR T cells: chimeric antigen receptor T cells
CTLA-4: cytotoxic T-lymphocyte-associated protein 4
ECM: extracellular matrix
HIF: hypoxia-inducible factor
IDO: indoleamine 2,3-dioxygenase
MDSCs: myeloid-derived suppressor cells
NSCLC: non-small cell lung cancer
PD-1: programmed cell death protein 1
PD-L1: programmed death-ligand 1
PGE2: prostaglandin E2
ROS: reactive oxygen species
SCLC: small cell lung cancer
STING: stimulator of interferon genes
TAAs: tumor-associated antigens
TAMs: tumor-associated macrophages
TME: tumor microenvironment
Tregs: regulatory T cells
VEGF: vascular endothelial growth factor
